# Understanding Medicine Preferences of Older Adults: The Role of Messaging in a Multi‐Methods Experimental Survey

**DOI:** 10.1111/hex.70366

**Published:** 2025-08-06

**Authors:** Alexander Chaitoff, Kristie Rebecca Weir, Vincent D. Marshall, Sarah E. Vordenberg

**Affiliations:** ^1^ University of Michigan School of Medicine Ann Arbor Michigan USA; ^2^ Leeder Centre for Health Policy, Economics, and Data, Sydney School of Public, Faculty of Medicine and Health University of Sydney Sydney Australia; ^3^ Institute of Primary Health Care (BIHAM) University of Bern Bern Switzerland; ^4^ University of Michigan College of Pharmacy Ann Arbor Michigan USA; ^5^ University of Michigan Center for Bioethics and Social Sciences in Medicine Ann Arbor Michigan USA

**Keywords:** deprescriptions, geriatrics, prescribing, survey

## Abstract

**Background:**

Efforts to optimise medicine use often focus on starting evidence‐based therapies or deprescribing inappropriate medicine, but not on the combination of both. We explored whether behavioural science‐informed messaging could impact older adults' decisions on starting and stopping diabetes medicine.

**Methods:**

A 15‐min online survey was conducted using a hypothetical vignette among adults 65 years and older from Australia and the United States. Participants were randomised in two experiments: the first involved different framings of a primary care provider (PCP)'s rationale for starting a diabetes medicine, and the second focused on stopping the same medicine 10 years later. Preferences were measured using 6‐point Likert scales. Data were analysed using descriptive and nonparametric statistics. We also conducted a descriptive thematic analysis of participants' free‐text responses explaining their preferences.

**Results:**

Out of 2420 participants, 68.5% agreed with starting the medicine, whereas 64.4% agreed with stopping it (*p* = 0.009). The proportion of participants who agreed with the recommendation to start or stop the medicine did not differ by the messaging they received in the first experimental treatment. However, participants were more likely to agree with a recommendation to stop a medicine if they received messaging about the medicine's harms versus lack of benefit (69.7% vs. 59.1%, *p* < 0.001). Thematic analysis revealed that participants' trust in clinician expertise, their perceptions of the medicine (e.g., benefits, harms, necessity and burdens) and a hesitance to change influenced their decisions.

**Conclusion:**

Two‐thirds of participants agreed with the recommendation to start and stop a diabetes medicine regardless of messaging at medicine initiation. Subsequent messaging focused on the potential harm of continuing a medicine significantly increased participants' agreement with deprescribing compared to messaging about the lack of benefit.

**Patient or Public Contribution:**

Older adult community members and general practitioners contributed to the development of the survey and vignette, helping to refine the content and improve its clarity and relevance.

## 1 Introduction

Medicine can be a double‐edged sword. This is especially true for older adults, a population more likely to qualify for evidence‐based medicine but also experience a higher likelihood of being harmed by those same treatments [1, 2]. Oftentimes, medicine that is initially the cornerstone for treating chronic disease become inappropriate when they are continued despite a patient no longer receiving benefits due to evolving age, frailty and health status [3, 4]. In this way, medicines that start as critical can become inappropriate and lead to significant quality and safety issues among older adults [5, 6].

Currently, efforts to address medicine use focus either on intervening at the time of prescribing to ensure evidence‐based therapy is started or on deprescribing chronically used inappropriate medicine, but not on how communication during both scenarios can affect comfort with the evolving need for medication use [7, 8]. It is accepted that the decision to start a medicine can depend on how the medicine is introduced [9]. However, only one study has examined how the introduction of a medicine affects patient attitudes towards discontinuation when the potential harms begin to outweigh the benefits [10]. This is important to consider, as language suggesting a treatment is ‘for life’ at the time of prescribing could create strong attachment and resistance to change, even when clinical circumstances evolve [11, 12, 13]. Despite this, conversations at the time of prescribing rarely include discussion of treatment duration, goals or plans for future reassessment [14, 15, 16]. Similarly, little is known about whether the framing of medicine use during conversations about stopping affects patients' preferences.

Addressing communication about medicine use is essential to fostering shared decision‐making and supporting safer, more appropriate prescribing over time [17]. Behavioural science‐informed messaging has shown promise as a low‐cost intervention to influence health‐related decision‐making in other contexts, such as vaccine uptake and cancer screening [18, 19, 20, 21, 22]. Techniques such as priming, which shapes individuals' preferences by exposing them to specific information, and prospect theory, which shapes individuals' actions by loss framing messages, may be useful for improving communication about medicine [23, 24]. The application of these approaches in supporting medicine optimisation and deprescribing decisions among older adults remains largely unexplored.

Diabetes is one chronic disease for which clinical guidelines recommend using medicine to achieve intense glycemic targets until a patient develops increasing frailty or limited life expectancy [25, 26]. Maintaining optimal blood glucose helps to avoid long‐term complications such as nephropathy, neuropathy and retinopathy [27]. Most Type 2 diabetes is diagnosed in middle‐aged adults, so starting medication is recommended for most patients [28]. However, especially as patients age, the same medicine can cause adverse effects, notably hypoglycemia, which can increase the risk of falls and fractures [29, 30]. Therefore, clinical guidelines recommend relaxing the haemoglobin A1c goal to < 8% among older adults with ‘significant cognitive and/or functional limitations, frailty, severe comorbidities, and a less favorable risk‐to‐benefit ratio of diabetes medicines’ and ‘avoid[ing] reliance on glycemic goals’ for older adults with very complex or poor health [25]. Unfortunately, many older adults remain on glucose‐lowering medicine despite HbA1c levels already being within guideline‐recommended thresholds, raising concerns about unnecessary treatment burden and the potential for harm [4, 31, 32, 33, 34]. Thus, diabetes serves as the ideal case for testing the hypothesis that behavioural science‐informed messaging at the time of prescribing and deprescribing can impact comfort with starting and stopping medicine for chronic diseases that require evolving amounts of treatment over time.

## 2 Methods

We conducted a 15‐min online survey using a hypothetical vignette with older adults recruited from Australia and the United States (US), reported in accordance with the CONSORT guidelines [35]. For Australia, the University of Sydney Human Research Ethics Committee granted ethics approval (2024/HE001357), and the University of Michigan Institutional Review Board deemed this study exempt for the US. It was registered at Clinicaltrials.gov with the ID NCT06698770 on 19 November 2024.

### 2.1 Study Design and Sample

Adults 65 years and older were recruited through an opt‐in panel of internet users in Australia via Dynata (Shelton, CT) and the US via Qualtrics Research Panels (Provo, UT) from November 2024 to January 2025. A random subset of eligible panellists were invited to participate according to our study's prespecified sample size (*n* = 1200 per country) and demographic distributions. A sample of size 2400 (*n* = 600 per arm) would allow us to detect a 10% difference in a dichotomous measure with power = 0.95 and *α* = 0.05. We included quotas to ensure a demographically diverse sample by age (50% 65–69 years and 50% 70 years and older), gender (50% female), education (70% less than a bachelor's degree) and race and ethnicity in the US sample (18% Hispanic, 15% Black, 5% Asian and 62% White or another race or ethnicity). The sampling algorithm invited panellists to complete the survey until the quotas were achieved. Strategies such as checking IP addresses, digital fingerprint technology and deduplication technology were used to prevent multiple responses by one participant. The survey invitation did not include the study topic to avoid self‐selection bias. Anonymous data collection was performed using Qualtrics software (Provo, UT). Participants were compensated based on the conditions of their panel agreement.

### 2.2 Survey Development

Our multidisciplinary research team included a physician (US), pharmacist (US) and health communication researcher (AUS) who collaboratively wrote a hypothetical vignette in which a doctor suggested starting a diabetes medicine (Part 1) and then suggested stopping it 10 years later (Part 2; Appendix A). Previous deprescribing surveys informed the initial draft of the hypothetical vignette [36, 37, 38]. The doctor was referred to as a primary care provider (PCP) in the US survey and a general practitioner (GP) in the Australian survey, but both are referred to as PCPs here.

We received feedback on the survey from approximately eight physicians and researchers specialising in risk‐communication from the University of Michigan Center for Bioethics and Social Sciences in Medicine Working Group. After incorporating these recommendations, we obtained feedback from a focus group of 16 older adult community members via the Michigan Institute for Clinical and Health Research Community Engagement Studio. The hypothetical vignette was then reviewed by seven physicians from Australia and Switzerland, along with five community members from Australia, to assess its clarity and clinical relevance. We subsequently pilot tested the full survey by distributing it to 50 participants per country within the Qualtrics Platform to verify that the survey flow and questions were being administered as planned, and checked the timing. We made minor updates to improve the clarity of Part 1, the starting medicine portion of the hypothetical vignette, and then proceeded with full survey administration.

### 2.3 Intervention

#### 2.3.1 Part 1: Starting a Diabetes Medicine

We created a vignette asking participants to imagine themselves as a patient during a primary care visit about managing diabetes. The vignette begins with the patient discussing efforts to improve their health and inquiring about their recent blood sugar levels. The PCP informs the patient that their haemoglobin A1c is 7.8%, which is higher than the goal of 7%. Participants were then randomised to receive one of four message combinations that related to both how the benefits of the medicine were communicated and how the duration of the medicine use was communicated (experimental Treatment 1). These scenarios included: control (neutral framing about medicine benefits, expected lifetime use of medicine), prospect alone (loss framing about medicine benefits, expected lifetime use of medicine), priming alone (neutral framing about medicine benefits, expected periodic re‐evaluation about medicine necessity) and prospect plus priming (loss framing about medicine benefits, expected periodic re‐evaluation about medicine necessity).

Below is the information provided to participants for each message.

- Neutral framing: ‘The good news is that taking diabetes medicine for your high blood sugar can help you significantly lower your chance of health problems in the future, like heart attacks and strokes’. We referred to this framing as ‘neutral’ given we presented the evidence‐based benefit of treatment (i.e., risk reduction) without explicitly invoking the negative consequences of inaction and did not highlight the level of risk associated with inaction.
- Loss framing: ‘Unfortunately, without diabetes medicine to lower your blood sugar, you will be at higher risk of having serious health problems in the future, like heart attacks and strokes’. This framing deliberately highlighted the negative consequences and high‐risk nature of the disease state.
- Lifetime use: ‘Based on research studies, I think that you will need to take this diabetes medicine for the rest of your life as high blood sugar is a chronic problem’.
- Lifetime use with periodic re‐evaluation: ‘Based on research studies, I think that you will need to take this diabetes medicine for the rest of your life. However, I always re‐evaluate the risks and benefits of any medicine I prescribe’.

#### 2.3.2 Part 2: Stopping a Diabetes Medicine

Participants were asked to imagine it is now 10 years later and they are visiting their PCP for a routine check‐up. They reported challenges checking their blood sugar at home due to increasing difficulty handling the test strips and reading the results. The PCP says their haemoglobin A1c is 7.2%, indicating well‐controlled blood sugar based on new research that supports different blood sugar goals at various life stages, and suggests adjusting to a more relaxed target of 8%. Participants were randomised to receive a rationale for stopping the medicine due to its lack of benefit or potential for harm, as shown below (experimental Treatment 2).

- Lack of benefit: ‘There comes a point where lower blood sugar may not stop things like heart attacks and strokes. However, a positive of this change is that you could stop checking your blood sugar at home’.
- Potential for harm: ‘As you age, you're at higher risk of eventually being harmed by your medicines. I worry that continuing your medicine could cause your blood sugar to go too low over time. This could lead to falls, bone fractures, and hospitalizations’.

### 2.4 Outcomes Measures

Our primary outcomes were related to agreement with the recommendation to start the diabetes medicine and to stop the diabetes medicine.

- *Agreement with starting the diabetes medicine*. The participants' attitude towards starting the diabetes medicine immediately after hypothetical vignette Part 1 as measured in response to the question, ‘What would you prefer to do with this diabetes medicine?’ on a 6‐point Likert scale with ‘Strongly prefer not to start medicine (1)’ and ‘Strongly prefer to start medicine (6)’ as the scale anchors. Participants were also asked to provide a free‐text rationale for their numerical response.
- *Agreement with stopping the diabetes medicine*. The participants' attitude towards stopping the diabetes medicine immediately after hypothetical vignette Part 2 as measured in response to the question, ‘What would you prefer to do with this diabetes medicine?’ on a 6‐point Likert scale with ‘Strongly prefer to continue (1)’ and ‘Strongly prefer to stop (6)’ as the scale anchors. Participants were also asked to provide a free‐text rationale for their numerical response.

### 2.5 Covariate Measures

To be able to describe our sample and consider effect‐modifying relationships between message framing and preferences, we gathered additional self‐reported information related to their history with diabetes, other health‐related characteristics and demographic characteristics.

#### 2.5.1 Diabetes‐Related Characteristics

- *Ever diagnosed with prediabetes or diabetes*. Participants were asked, ‘Has a healthcare professional ever told you that you have pre‐diabetes or diabetes?’ with response options of ‘yes’, ‘no’ and ‘I don't know’.
- *Current type of diabetes*. Participants who indicated that they were ever diagnosed with prediabetes or diabetes were asked, ‘What type of diabetes do you currently have?’ with response options of ‘prediabetes’, ‘Type 1’, ‘Type 2’, ‘a different type of diabetes’, ‘I don't know’ and ‘I do not currently have any type of prediabetes or diabetes’.
- *Current control of Type 1 and Type 2 diabetes*. Participants with Type 1 or Type 2 diabetes were asked, ‘How well controlled is your diabetes?’ with response options ‘Very well controlled’, ‘Somewhat well controlled’, ‘Not very well controlled’ and ‘I do not know’.
- *Diabetes medicine*. Participants who reported a diabetes diagnosis were asked whether they currently, previously or never took diabetes medicine.

#### 2.5.2 Other Health‐Related Characteristics

- *Support to manage medicine*. Participants were asked whether they needed ‘minimal support’, ‘occasional support’ or ‘complete assistance’ to manage their medicine.
- *Self‐reported health*. Participants' perceptions of their health as measured by their response to the question, ‘In general, how would you rate your health today?’ with responses ranging from ‘Poor (1)’ to ‘Excellent (5)’ [39].
- *Health literacy*. Participants' confidence in filling out medical forms as measured using a single item with responses ranging from ‘Not at all (1)’ to ‘Excellent (5)’ [40, 41].
- *Number of medicines*. Participants reported the number of prescription medicines and non‐prescription medicines and/or dietary supplements they take in a typical week.

#### 2.5.3 Demographic Characteristics

- These included participants' country of residence, gender, highest level of education and age.

### 2.6 Data Analysis

To summarise our data, we used descriptive statistics, count, percent, median and interquartile range (IQR). These were chosen due to the skewness of the distributions. To assess the balance of covariates between those randomised to the four scenarios in experimental Treatment 1 and the two scenarios in experimental Treatment 2, we further used Pearson's chi‐square tests for categorical variables and Mann–Whitney *U* and the Kruskal–Wallis test for numeric variables.

We considered agreeing to start and agreeing to stop the diabetes medicine as two separate binary outcomes (coded as agree when participants answered 4–6 on each Likert scale, respectively). We used chi‐square tests to assess the impact of being randomised to control, prospect theory‐only, priming‐only or both prospect theory and priming messaging in experimental Treatment 1 on participants' agreement regarding both starting and stopping the diabetes medicine, respectively. Doing this, we were assessing if the messaging at the time of prescribing was associated with both agreement related to starting and subsequently stopping the medicine. Next, we used chi‐square tests to assess the impact of being randomised to a harm or lack of benefit messaging scenario in experimental Treatment 2 on participants' agreement with stopping the medicine. When a significant association between experimental treatment and participant agreement existed, we conducted subgroup analyses by assessing the interaction between experimental treatment variable and our covariates to report the odds ratio, 95% confidence interval and *p*‐value of the interaction. Of note, we limited our analysis to a predefined set of participant characteristics we felt could be conceptually linked to medication preferences rather than looking at associations between every possible participant characteristic and agreement, which also limited the risk of a Type 1 error. Finally, we used McNemar's test to compare participants' agreement regarding starting versus stopping the diabetes medicine. We considered an alpha of 0.05 to be our Type I error rate, and all tests were two‐sided. All analyses were conducted on R statistical software, v 4.2.2 [42]. Interaction contrasts were performed with the emmeans package, and the forest plots were constructed with the forestplot package [43, 44].

We also conducted a descriptive thematic analysis of participants' free‐text responses explaining their reasons for starting and subsequently stopping the diabetes medicine. Following an inductive approach, one researcher read through all responses, identifying preliminary patterns and themes present across the two decision points (starting and stopping a medicine), discussing the findings with another researcher. As the analysis progressed, it became apparent that similar themes were present at both decision points (starting and stopping the medicine). After identifying the main themes, free‐text responses at both decision points were reanalysed for confirmation. Example quotes were selected to illustrate each theme. Quantitative counts of theme frequency were not performed, consistent with the descriptive purpose of the analysis.

## 3 Results

**Figure 1:**
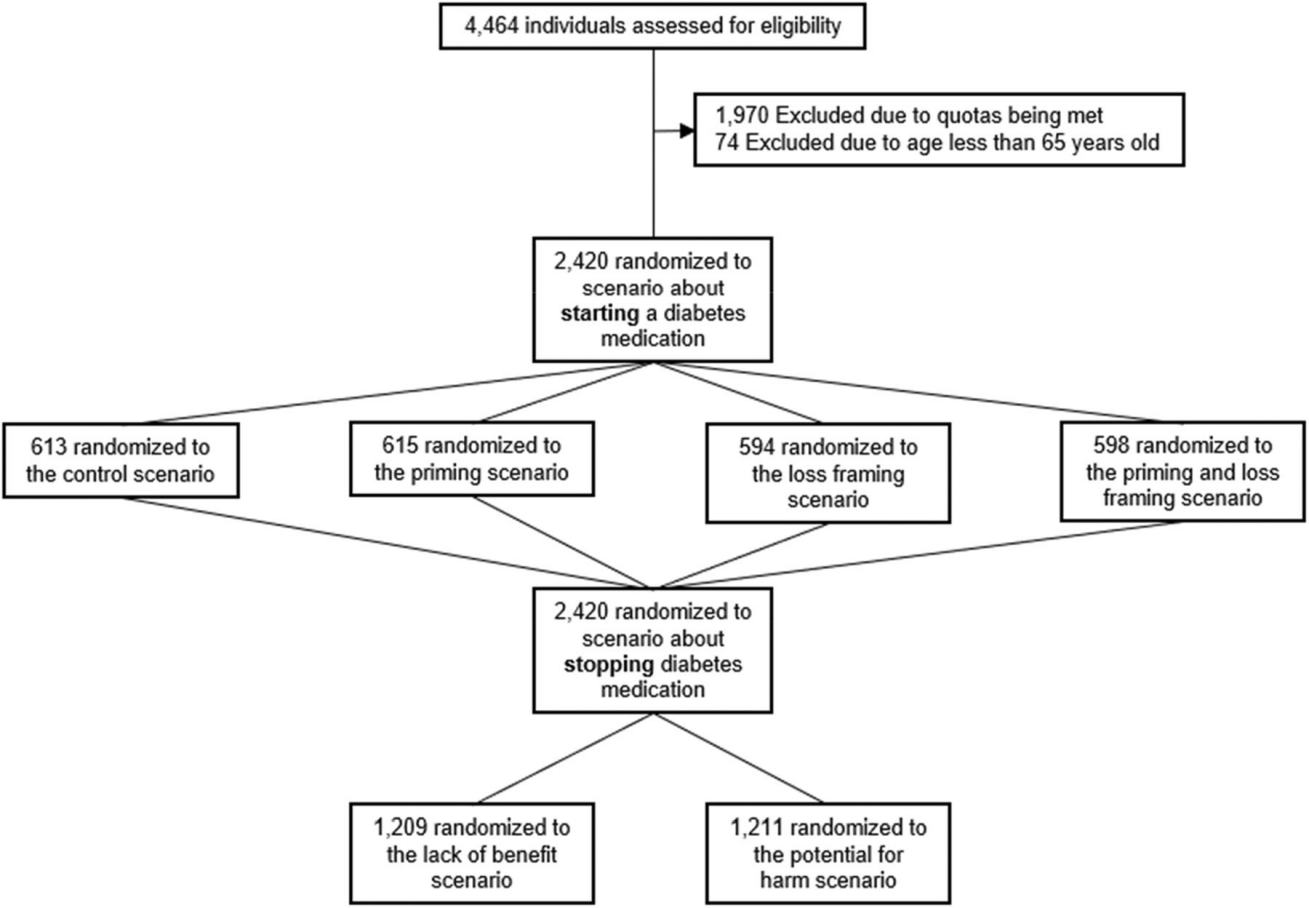
Participant flow and study design.

Of the 4464 individuals who entered the survey, 1970 were excluded due to sampling quotas for their demographic group already being filled, and 74 were excluded for self‐reporting being < 65 years (Figure 1). This yielded 2420 participants (*n* = 1202 from Australia and *n* = 1218 from the US) in the final analytic sample, all of whom completed the survey. The sample had a median age of 70 (IQR 67–74), and 48.3% (*n* = 1169) of participants were female (Table 1). Approximately one‐third of participants reported having received a diagnosis of prediabetes or diabetes (32.9%, *n* = 796). Participants self‐reported being prescribed a median of 3 (IQR 1–5) medicines, and 80.1% (*n* = 1939) reported requiring minimal support to manage their medicine. There were no clinically or statistically meaningful differences between those randomised to any of the four experimental starting scenarios (Appendix B) or between those randomised to either of the two experimental stopping scenarios (Appendix C).

**Table 1:** Older adults' demographic characteristics and clinical experiences (*n* = 2420).

	Number (%)
*Demographic characteristics*	
Country of residence	
Australia	1202 (49.7)
United States	1218 (50.3)
Gender	
Male	1246 (51.5)
Female	1169 (48.3)
Another gender or not provided	5 (0.2)
Education	
High school diploma or less	713 (29.5)
Some college, associate's or trade school	905 (37.4)
Bachelor's degree	513 (21.2)
Master's degree or higher	289 (11.9)
*Diabetes‐related characteristics*	
Ever diagnosed with prediabetes or diabetes	796 (32.9)
Current type of diabetes	
Prediabetes	221 (9.1)
Type 1	34 (1.4)
Type 2	453 (18.7)
Do not currently have diabetes	67 (2.8)
Do not know the type	21 (0.9)
Current control of Type 1 and Type 2 diabetes	
Very well	214 (8.8)
Somewhat	237 (9.8)
Not very	30 (1.2)
Not sure	6 (0.2)
Diabetes medicine	
Current	503 (20.8)
Previous	64 (2.6)
Never	1637 (67.6)
*Other health‐related characteristics*	
Support to manage medicine	
Minimal support	1939 (80.1)
Occasional support	204 (8.4)
Complete assistance	61 (2.5)
Self‐reported health	
Excellent	102 (4.2)
Very good	591 (24.4)
Good	1078 (44.5)
Fair	558 (23.1)
Poor	91 (3.8)
Health literacy	
Extremely	1468 (60.7)
Quite a bit	657 (27.1)
Somewhat	210 (8.7)
A little bit	41 (1.7)
Not at all	44 (1.8)

	**Median (IQR)**
Age	70 (67–74)
Total number of medicines	4 (2–7)
Prescription medicines	3 (1–5)
Over‐the‐counter medicines and supplements	1 (0–3)

**Figure 2:**
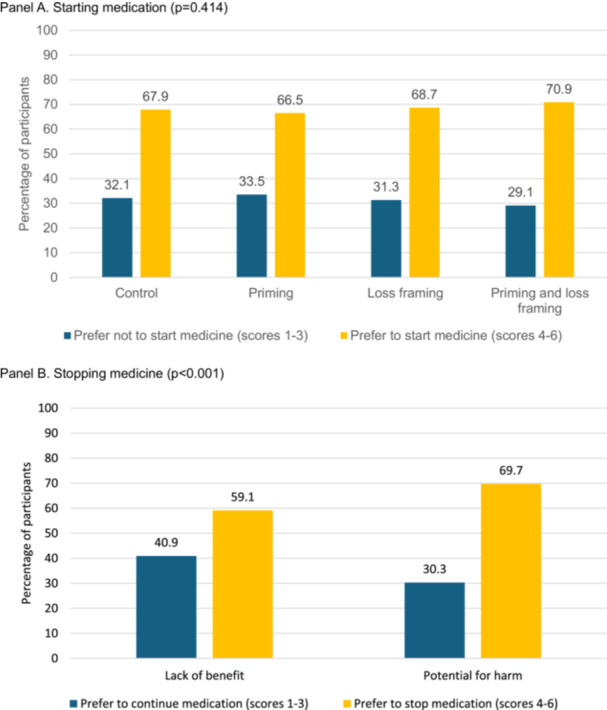
Older adults' preferences regarding starting (A) and subsequently stopping (B) the diabetes medicine by hypothetical vignette (*n* = 2420). (A) Starting medicine (*p* = 0.414). (B) Stopping medicine (*p* < 0.001).

**Figure 3:**
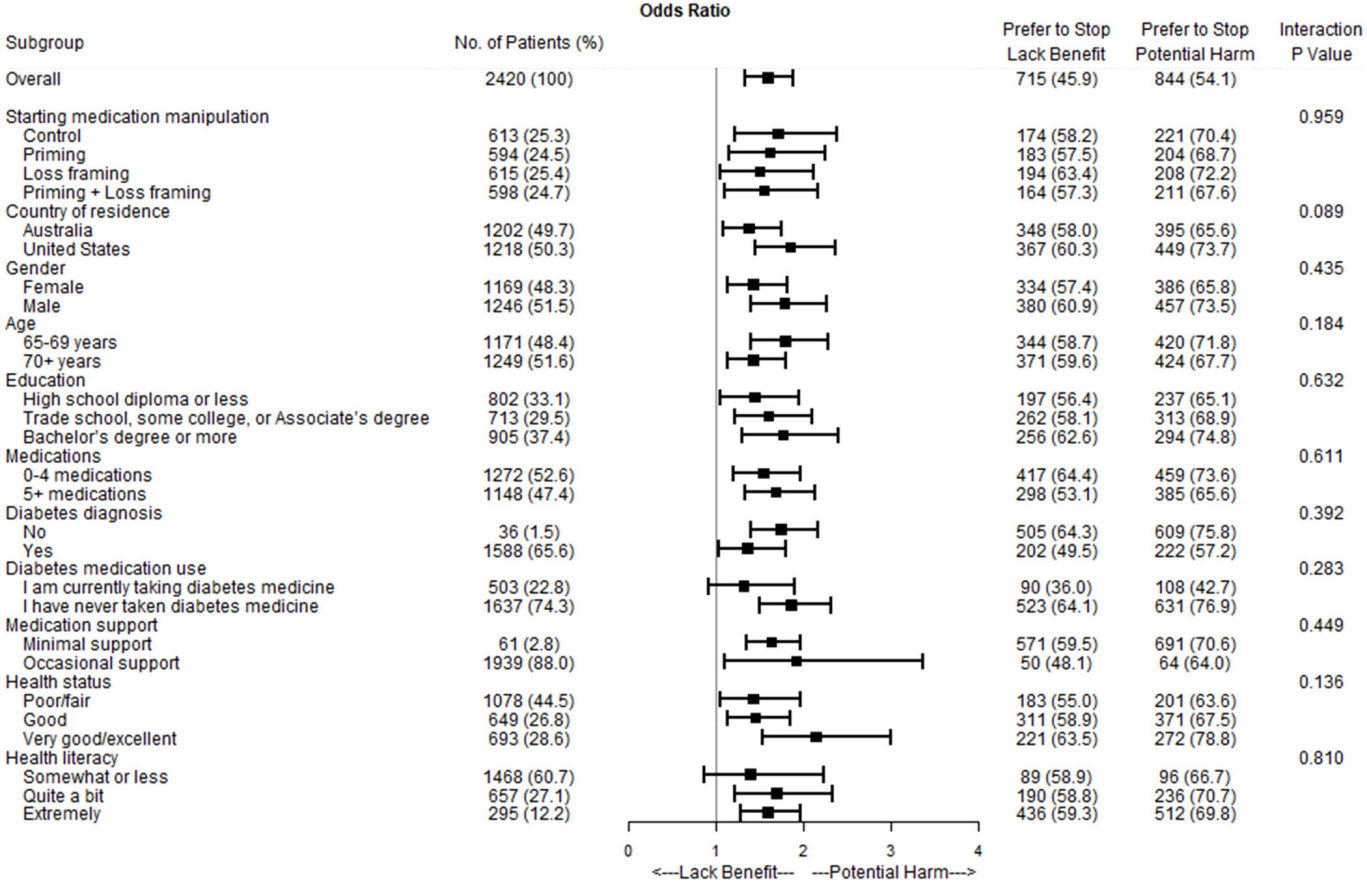
Older adults' agreement with deprescribing by rationale for medicine discontinuation.

Participants were more likely to agree with starting the diabetes medicine (*n* = 1657, 68.5%) than stopping it (*n* = 1559, 64.4%; *p* = 0.009). The proportion of participants who agreed with the starting recommendation did not differ by which scenario they were randomised to in experimental Treatment 1 (Figure 2A). Similarly, the scenario to which they were randomised in experimental Treatment 1 was not associated with the proportion that agreed with the recommendation to stop the medicine (Figure 3). However, the proportion of participants who reported they agreed with the recommendation to stop the medicine was significantly higher among those randomised to receive messaging about the medicine's potential for harm (69.7%) versus those randomised to view messaging about the medicine's lack of benefit (59.1%) in experimental Treatment 2 (*p* < 0.001, Figure 2B). This finding was consistent across subgroups.

Several patterns were identified during the thematic analysis of participants' free‐text responses explaining their rationale for preferring starting or stopping the diabetes medicine. These included thoughts on the relationship with the clinician, perceptions about medicine and hesitance to change (Table 2). Many participants reported deference to the expertise of the clinician, with some reporting they preferred being instructed rather than being asked to contemplate medicine decisions. Irrespective of whether participants were considering starting or stopping a medicine, many participants indicated a preference to avoid starting a medicine, including explicitly stating they would like lifestyle options to consider. This wish to avoid medicine led to diverging opinions about being told they could stop taking a medicine. That is, some participants questioned why the medicine was no longer beneficial, whereas others reported frustration, now believing they had been told the medicine was never beneficial.

**Table 2:** Older adults' reflections when considering starting and subsequently stopping a diabetes medicine.

Theme	When considering starting the medicine	When considering stopping the medicine
*Clinician‐related*		
Trust in the doctor's recommendation	Participants described trust in the doctor's recommendation to start the medicine and to follow medical advice, *‘I would start because my doctor recommended that I do’*.	Participants expressed a reliance on their doctor's judgement and to follow their doctor's advice whether that meant continuing, *‘doctor recommends continuing’* or stopping the medicine, *‘efficacy is low, doctor is counselling to stop as the better alternative’*. Or leave the decision entirely to the doctor, *‘I'd prefer for the GP to just tell me whether or not to stop, he's the doctor and that's his decision to make in my mind’*.
*Medicine‐related*		
Perceived health benefits	Participants described expected benefits such as stabilising blood sugar, staying healthy and preventing future complications, *‘To possibly avoid heart or other serious problems’*.	Participants wanted to continue the medicine because it works well, maintains their health and prevents complications, *‘After taking the medicine, my blood sugar has been well controlled, all indicators are very stable, and there are almost no obvious side effects. I hope to continue using it*’.
Harms of the medicine	Participants had concerns about potential harms or side effects of the medicine, negative views of long‐term medicine use, *‘I would prefer not to start. I feel like I become a prisoner to the medicine’*.	Similarly, participants wanted to avoid harms or side effects of the medicine, especially with older age or long‐term use, *‘If it could be harmful in the long run, I don't want to take it’*.
Lack of necessity of the medicine	While few participants said the medicine was completely unnecessary, some questioned whether the medicine was needed, expressing doubts about its effectiveness, *‘it only helps 4 more people out of the 100’*, and a preference to manage their condition in other ways first, they'd rather *‘try harder on my own… before giving in to what might become a lifetime requirement’*.	Participants questioned the continued need for the medicine, particularly when it no longer provided clear benefits or was no longer aligned with the revised treatment goals, *‘I feel like it is not necessary as I am within today's guidelines’*. Some preferred to avoid medicine altogether, *‘Less medication the better, especially when it's unnecessary’*.
Monitor, reduce, trial, assess	A few participants preferred a stepwise approach to starting the medicine, beginning with a low dose, monitoring its effects and adjusting based on outcomes. One would *‘start on a lower level then increase as needed’*, whereas another would *‘start it and see what the blood tests reveal’*.	Participants preferred to trial reducing the dose, taper slowly, monitor and review the medicine, *'I would like to gradually slow down on taking the medicine. Would just like to see how it would be at the next PCP visit’* and re‐evaluate if needed, *‘Continue for a while and monitor results and then perhaps stop’*.
Burden of medicine	A few participants mentioned the burden of taking multiple medicines, *‘I take enough medicines already’* and that the patient in the scenario *‘seems overwhelmed with all the things to do’*.	Participants described the practical and emotional burden of managing their medicine and monitoring routines. Challenges included cost, *‘it is a pain and not cheap’*, physical discomfort, *‘the machine hurts my fingers’* and the effort required to keep track of treatment tasks, *‘Just one thing less I have to keep track of’*.
Preference for lifestyle changes and self‐management	Participants expressed a preference for managing their diabetes through lifestyle changes, such as diet and exercise, reassessing if the medicine is needed, *‘I would stop eating foods loaded with sugar and later have a blood test to check results’*.	Participants expressed a similar preference for self‐management strategies including diet and lifestyle to avoid relying on medicine, *‘If my sugar went too low that would not be good either, I would rather stop and change my diet and get more exercise, than to stay on meds’*.
*Hesitancy towards change*		
Need for more information	Participants felt they needed more information, time to think, to do their own research or seek a second opinion before deciding, *‘I would like to seriously give it some thought, do some reading than decide’*.	Similarly, participants expressed feeling undecided, needing more information or a second opinion before deciding, *‘I would need to know more about the pros and cons to make an informed decision’*, and another said, *‘Undecided, I might want to continue to help prevent future health issues. Or find out why not’*.
Preference for stability	N/A	Participants preferred to continue their medicine to maintain stability and avoid disrupting a routine that works, *‘Reluctant to make a major change to a system that has worked efficiently’*. They described feeling comfortable with the current regimen, *‘feeling OK so keep doing the same thing’*, and viewed change as potentially risky: *‘I think continuing on is the better and safer option’*.
Doubt about the initial need for medicine	N/A	Participants expressed doubt and frustration towards the scenario and doctor about whether the medicine was ever needed, *‘I'd be concerned with the change in attitude and direction by the doctor’*. Changing clinical advice and evolving treatment targets led some to question the original decision to start the medicine and the credibility of the information they had received, *‘Why was I on this for so long and now you say 8% is acceptable?’*. This raised broader concerns about trust, communication and the quality of care, *‘All the “relax approach” means that I've taken medication for 10 years without needing it!’*

## 4 Discussion

In this vignette‐based experimental survey, approximately two‐thirds of participants agreed with starting and subsequently stopping a diabetes medicine, with no significant differences across behavioural science‐informed messaging at the time of prescribing. However, at the time of deprescribing, participants who were presented with information highlighting the potential harm of continuing the medicine were significantly more likely to agree with stopping it compared to those told about a lack of benefit. Participants' free‐text responses revealed that important factors in their decision‐making process included trust in the PCP's expertise, their perceptions of the medicine (e.g., benefits, harms, necessity and burdens) and a hesitance to change.

This study builds on a randomised vignette‐based online experiment in which older adults in the US were asked to imagine starting a proton pump inhibitor (PPI) for heartburn [31]. Those who received warning information about long‐term harms at the time of medicine initiation were significantly more willing to stop the medicine when their PCP later recommended it. Here we found that over one‐third of older adults were interested in deprescribing a diabetes medication at the recommendation of their PCP. Despite this, deprescribing guidelines rarely define for whom medicines are no longer helpful and in whom the risks are more likely to outweigh the benefits. This contrasts with prescribing guidelines, where high‐risk patients are clearly defined with numeric cut‐offs. Our results suggest a significant minority of patients are interested in deprescribing, and one step towards acting on that may be more concrete guideline recommendations.

We also found that older adults incorporated information about potential harms into their decision to stop the diabetes medications, as evidenced by 10.6% more participants preferring to stop the medicine when they received the potential for harm, as opposed to a lack of benefit justification for deprescribing. This suggests that clinical guidelines should not only provide general information about when and with whom to deprescribe but also emphasise the importance of prescribers communicating information about potential harms during deprescribing conversations. Furthermore, deprescribing shared decision‐making resources should emphasise information related to the potential for harm of stopping and continuing the medication.

Our hypothetical vignette mirrors a common situation faced by adults being treated for diabetes. We selected this format for our experiment because older adults have reported enjoying making medicine decisions within the context of hypothetical vignettes [45], and it is an efficient way to test hypotheses before investing significant resources and exposing patients to interventions in clinical settings. Although the vignette was specifically focused on diabetes, the results we report may have broader implications for chronic disease management in which there is a need to re‐evaluate treatments when a patient's health status evolves over time. Based on the free‐text responses in our study, some patients may have difficulty understanding why a treatment goal has changed, as evidenced by some participants questioning whether the medicine was initially appropriate to recommend.

Schoenborn et al. identified that the most important factor for older adults with Type 2 diabetes when they consider starting or stopping a diabetes medicine is the likelihood of adverse effects, as opposed to the disease duration, complications, other health conditions, life expectancy, treatment burden or cost [46]. Our study provides further support for these findings. This aligns with a previous hypothetical vignette study, where participants were more likely to agree to stop a statin to prevent cardiovascular events or a proton pump inhibitor for heartburn when informed that continuing the medicine could be harmful, compared with when they were told there was a lack of benefit with continued use [31]. This growing body of evidence suggests that older adults across multiple countries with various chronic conditions are influenced when provided information about potential medicine‐related harms. Additional research is needed to understand what type of harm information (e.g., common side effects, rare but serious side effects) is most meaningful to older adults when they are considering starting or stopping a medicine.

We found that behavioural science‐informed messaging at the time of prescribing did not influence participants' agreement with starting a diabetes medicine when it was appropriate and also did not impact their agreement with stopping a medicine when it was no longer appropriate. Our null findings regarding the impact of messaging at the time of prescribing on either preference could be because our messages lacked strength (i.e., wording was subtle or nuanced). Additional research is needed to determine whether different behavioural science‐informed messages or stronger language at the time of prescribing may influence medicine‐related preferences. Future work may also assess if harm‐related messaging at the time of prescribing and at the time of deprescribing have additive or multiplicative effects.

While medication decisions faced by older adults are often preference‐sensitive, many deprescribing studies focus on the more clear‐cut decisions. A strength of our study is that it addressed a somewhat preference‐sensitive clinical scenario, especially at the time of deprescribing, allowing participants to express their preferences about starting the medicine and, subsequently, continuing or stopping it. Also, we analysed participants' free‐text responses to understand the meaning behind the quantitative findings. The themes that were identified align with previous content analyses exploring factors important to older adults when they consider deprescribing a chronic medicine [13, 47]. For example, participants frequently have reported following their doctor's recommendation as they trust their expertise. Conversely, some participants would prefer to consider alternatives to medicine, such as lifestyle changes, to manage their health condition. This is particularly relevant for a condition like Type 2 diabetes given clinical guidelines emphasise lifestyle management as an important strategy for preventing and managing diabetes [48].

### 4.1 Limitations

The primary limitation of this study is the hypothetical vignette may not reflect real‐life decisions, especially given the imagined 10‐year gap between starting and stopping the diabetes medicine. Although we measured participants' preferences about starting the medicine, all were told the patient chose to start it, regardless of their initial response. To test the behavioural science messages, we intentionally used a ‘diabetes medicine’ that required regular home monitoring even though this may not fully reflect clinical practice guidelines. Although our sample included substantial demographic diversity, we acknowledge that it is not representative, given that all participants were willing and able to participate in an online survey. Though it was reassuring that approximately one‐third of older adults in this study reported prediabetes or diabetes, which is similar to the prevalence of the condition in Australia and the US [49, 50]. Finally, it is possible that additional demographic characteristics may be associated with willingness to deprescribe; however, we recognise that variables, such as race and ethnicity, are socially constructed proxies for social determinants and observing an association between these variables and willingness to deprescribing provides limited actionable insight and carries a risk of misinterpretation. Future work with larger, prospectively powered samples could investigate a broader array of social and structural determinants of deprescribing decisions.

## 5 Conclusion

Messaging at the time of deprescribing that focused on the potential harm of continuing a medicine significantly increased participants' preference for deprescribing compared to messaging about the lack of benefit. These results highlight that messaging can influence patient preferences, but its ability to do so is likely time and content‐dependent.

## Author Contributions

**Alexander Chaitoff:** conceptualization, methodology, statistical guidance, writing – original draft, writing – review and editing. **Kristie Rebecca Weir:** conceptualization, methodology, writing – original draft, writing – review and editing, funding. **Vincent D. Marshall:** formal analysis, writing – review and editing. **Sarah E. Vordenberg:** conceptualization, methodology, formal analysis, writing – review and editing, funding, supervision.

## Ethics Statement

For Australia, the University of Sydney Human Research Ethics Committee granted ethics approval (2024/HE001357), and the University of Michigan Institutional Review Board deemed this study exempt for the United States. It was registered at Clinicaltrials.gov with the ID NCT06698770.

## Conflicts of Interest

The authors declare no conflicts of interest.

## Data Availability

The data that support the findings of this study are available upon request from the corresponding author. The data are not publicly available due to privacy or ethical restrictions. The study was registered at Clinicaltrials.gov with the ID NCT06698770.

## Appendix A

**Hypothetical Vignette Displayed to Older Adults in the Online Survey**

*Part 1. Start a diabetes medicine*

On the next few pages, we will ask you to imagine that you are seeing your PCP (GP) for a routine medical visit. A PCP (GP) is your doctor who sees people for common medical conditions. Then, we will ask you questions about the scenario.

See Table A1.

**Table A1:** When reading the scenario and answering the questions, please think of yourself as the patient in this situation.

Control	Loss framing only	Priming: Future re‐evaluation	Loss framing + priming: Future re‐evaluation
A. **Your PCP/GP:** Good morning! How have you been feeling lately?			
B. **You:** Good morning. I'm pretty good. Since my last visit with you, I met with a dietician. I have been eating healthier and exercising. I am trying to keep my blood sugar from getting too high. I had blood tests yesterday. How was my blood sugar?			
Page break			
C. **Your PCP/GP:** It's great to hear you have made those healthy changes to manage your diabetes. The blood test measured your haemoglobin A1c. Haemoglobin A1c can tell your average blood sugar over the past 2–3 months. We usually want your haemoglobin A1c to be less than 7%. Yours was 7.8%. This means your blood sugar is still higher than what we typically like to see.			
D. **You:** That is disappointing. I remember that you gave me a health pamphlet at my last visit. It said that high blood sugar causes problems throughout the body. Many people even die from diabetes.			
E. **Your PCP/GP:** Diabetes can lead to problems like heart disease, nerve damage, kidney disease and blindness.			
F. **You:** That's worrying.			
Page break			
Ga. **Your PCP/GP: The good news is that taking diabetes medicine for your high blood sugar can help you significantly lower your chance of health problems in the future, like heart attacks and strokes.**	Gb. **Your PCP/GP: Unfortunately, without diabetes medicine to lower your blood sugar, you will be at higher risk of having serious health problems in the future, like heart attacks and strokes.**	Ga. **Your PCP/GP: The good news is that taking diabetes medicine for your high blood sugar can help you significantly lower your chance of health problems in the future, like heart attacks and strokes.**	Gb. **Your PCP/GP: Unfortunately, without diabetes medicine to lower your blood sugar, you will be at higher risk of having serious health problems in the future, like heart attacks and strokes.**
Page break			
H. **You:** What do you recommend?			
I. **Your PCP/GP:** I recommend starting a **pill that can lower your blood sugar** and your risk of more severe health problems.			
J. **You:** Okay, but are there any risks or side effects?			
K. **Your PCP/GP:** Most people tolerate the medicine well. However, like with any medicine, there are some potential side effects. Your doctor tells you about the benefits and potential side effects of the diabetes medication:			
**Benefits**		**Harms**	
4 fewer people out of 100 people have a serious health problem		Low blood sugar Weight gain Stomach upset Diarrhoea	
Page break			
L. **You:** How will I know if I have low blood sugar?			
M. **Your PCP/GP:** I would like you to **test your blood sugar two times a day** to make sure the medication is working well. It involves pricking your finger with a small needle to get a drop of blood. After you get the results, please write down the number. Bring your paper to your next appointment so I can see if the dose is just correct, too high or too low for you. In addition, you should test your blood sugar if you feel symptoms like tiredness, confusion, sweating and a racing heart. This could mean that you may have low blood sugar. If this happens, you should eat a small snack to raise your blood sugar.			
N. **You:** Ok. I am nervous about checking my blood sugar as it seems like it will hurt. But I will do it.			
Page break			
O. **Your PCP/GP:** We'll start with a low amount to see how your body reacts to it. You'll just need to get the medicine from your usual pharmacy and remember to take it daily. I'll also schedule a follow‐up appointment to check your blood sugar and adjust the amount if needed. **Please bring your blood sugar readings from home to the visit.**			
P. **You:** Wow, it sounds a lot. However, I am sure I can do it.			
Q. **Your PCP/GP:** Do you have any other questions?			
R. **You:** How long will I need to take this medicine?			
Page break			
Sa. **Your PCP/GP:** Most people need this medicine for a long time. **Based on research studies, I think that you will need to take this diabetes medicine for the rest of your life as high blood sugar is a chronic problem.** It is also important to live a healthy lifestyle by eating a healthy diet, exercising, not smoking and keeping a healthy weight. **I encourage you to keep up those healthy habits, as it may allow you to stop taking the medicine in the future.**		Sb. **Your PCP/GP:** Most people need this medicine for a long time. **Based on research studies, I think that you will need to take this diabetes medicine for the rest of your life. However, we'll plan to review your medication regularly to check if it's working well for you and see if any changes would be helpful.** It is also important to live a healthy lifestyle by eating a healthy diet, exercising, not smoking and keeping a healthy weight. **I encourage you to keep up those healthy habits, as it may allow you to stop taking the medicine in the future.**	
T. **You:** That makes sense. I don't have any other questions.			
U. **Your PCP/GP:** I'll send the prescription to the pharmacy. I'll see you at your next appointment.			
V. **You:** Thank you.			

Based on what you have read so far, **what would you prefer to do with this diabetes medicine?** [6‐point Likert scale with scale anchors ‘Strongly prefer **not** to start medicine’ (1) and ‘Strongly prefer to **start** medicine’ (6)].

*Part 2. Stopping a diabetes medicine*

Imagine that you decided to start the diabetes medicine based on the recommendation of your PCP/GP. You do not experience any side effects or problems when starting the medicine.

[Page break]

Imagine it is now **10 years later**. You visit your PCP/GP for a routine medical visit. On the following pages, you will see a conversation between you and your PCP/GP.

[Page break]

See Table A2

**Table A2:** When reading the scenario and answering the questions, please think of yourself as the patient in this situation.

Reason for deprescribing = lack of benefit	Reason for deprescribing = potential for harm
A. **Your PCP/GP:** Good morning! How have you been feeling lately?	
B. **You:** Good morning. Every time I see you I feel like life is getting a little harder. My age is really catching up with me. It's been more challenging to keep up with everything that I need to do to care for myself and my home. **Things are getting harder.**	
C. **Your PCP/GP:** Thank you for sharing that with me. Is there anything in particular that you would like to discuss today about your health or caring for yourself and your home?	
Page break	
D. **You: It's been harder for me to check my blood sugar at home.** It's difficult to put the test strip into the machine, and I have a hard time seeing the results. I did go to the lab yesterday though. I am curious about the results.	
E. **Your PCP/GP:** I'm glad you brought that up. The test you use at home looks at your blood sugar at the moment. The test we conduct in the lab looks at haemoglobin A1c, which looks at your average blood sugar over the past 2–3 months. Today, it was 7.2%. I think that your blood sugar is well‐controlled.	
F. **You:** That's great news, but I always thought that you said my haemoglobin A1c should be less than 7%.	
G. **Your PCP/GP:** Research these days suggests that there should be different blood sugar goals at different times in people's lives. I think it may be time to adjust your diabetes medicine to align with a more relaxed blood sugar goal of 8%.	
Page break	
H. **You:** Why do you recommend a more relaxed blood sugar goal?	
Ia. Your PCP/GP: **There comes a point where lower blood sugar may not stop things like heart attacks and strokes. However, a positive of this change is that you could stop checking your blood sugar at home.**	Ib. Your PCP/GP: **As you age, you're at higher risk of eventually being harmed by your medicines. I worry that continuing your medicine could cause your blood sugar to go too low over time. This could lead to falls, bone fractures and hospitalisations.**
J. **You:** Ok.	
K. **Your PCP/GP: What do you think about stopping your diabetes medicine?**	

Based on what you have read so far, **what would you prefer to do with this diabetes medicine?** [6‐point Likert scale with scale anchors ‘Strongly prefer to **continue**’ (1) and ‘Strongly prefer to **stop**’ (6)].

## Appendix B

See Table B1.

**Table B1:** Older adults' demographic characteristics and clinical experiences when starting a medicine by hypothetical vignette (*n* = 2420).

	Number (%)				*p* value
	Control (*n* = 613)	Priming (*n* = 615)	Loss framing (*n* = 594)	Priming and loss framing (*n* = 598)	
*Demographic characteristics*					
Country of residence					
Australia	299 (48.8)	304 (49.4)	299 (50.3)	300 (50.2)	0.948
United States	314 (51.2)	311 (50.6)	295 (49.7)	298 (49.8)	
Gender					
Male	322 (52.5)	303 (49.3)	321 (54.0)	300 (50.2)	0.472
Female	291 (47.5)	310 (50.4)	271 (45.6)	297 (47.9)	
Another gender or not provided	0 (0)	2 (0.3)	2 (0.3)	1 (0.2)	
Education					
High school diploma or less	169 (27.6)	178 (28.9)	173 (29.1)	193 (32.3)	0.588
Some college, associate's or trade school	242 (39.5)	233 (37.9)	213 (35.9)	217 (36.3)	
Bachelor's degree or higher	202 (33.0)	204 (33.2)	208 (35.0)	188 (31.4)	
*Diabetes‐related characteristics*					
Ever diagnosed with prediabetes or diabetes	200 (32.6)	219 (35.6)	171 (28.8)	206 (34.4)	0.152
Current type of diabetes					
Prediabetes	57 (9.3)	60 (9.8)	43 (7.2)	61 (10.2)	0.579
Type 1	7 (1.1)	11 (1.8)	5 (0.8)	11 (1.8)	
Type 2	114 (18.6)	127 (20.7)	99 (16.7)	113 (18.9)	
Do not currently have diabetes	16 (2.6)	15 (2.4)	18 (3.0)	18 (3.0)	
Do not know type	6 (1.0)	6 (1.0)	6 (1.0)	3 (0.5)	
Current control of Type 1 and Type 2 diabetes					
Very well controlled	52 (8.5)	65 (10.6)	46 (7.7)	51 (8.5)	0.750
Somewhat well controlled	59 (9.6)	67 (10.9)	50 (8.4)	61 (10.2)	
Not very well‐controlled	8 (1.3)	5 (0.8)	7 (1.2)	10 (1.7)	
Not sure	2 (0.3)	1 (0.2)	1 (0.2)	2 (0.3)	
Diabetes medicine					
Current	122 (19.9)	141 (22.9)	116 (19.5)	124 (20.7)	0.473
Previous	20 (3.3)	17 (2.8)	11 (1.9)	16 (2.7)	
Never	426 (69.5)	405 (65.9)	409 (68.9)	397 (66.4)	
*Other health‐related characteristics*					
Support needed to manage medicine					
Minimal support	505 (82.4)	495 (80.5)	477 (80.3)	462 (77.3)	0.231
Occasional support	51 (8.3)	57 (9.3)	43 (7.2)	53 (8.9)	
Complete assistance	12 (2.0)	11 (1.8)	16 (2.7)	22 (3.7)	
Self‐reported health					
Excellent or very good	183 (29.9)	165 (26.8)	173 (29.1)	172 (28.8)	0.800
Good	277 (45.2)	273 (44.4)	266 (44.8)	262 (43.8)	
Fair or poor	153 (25.0)	177 (28.8)	155 (26.1)	164 (27.4)	
Health literacy					
Extremely	374 (61.0)	365 (59.3)	374 (63.0)	355 (59.4)	0.593
Quite a bit	170 (27.7)	164 (26.7)	151 (25.4)	172 (28.8)	
Somewhat or less	69 (11.3)	86 (14.0)	69 (11.6)	71 (11.9)	

	**Median (IQR)**				
Age	70 (67–74)	69 (67–75)	70 (67–74)	70 (67–75)	0.478
Total number of medicines	4 (2–7)	5 (2–8)	4 (2–7)	4 (2–7)	
Prescription medicines	3 (1–5)	3 (1–5)	3 (1–5)	3 (1–5)	0.229
OTC medicines and supplements	1 (0–2)	1 (0–3)	1 (0–3)	1 (0–3)	

## Appendix C

See Table C1.

**Table C1:** Older adults' demographic characteristics and clinical experiences when stopping a medicine by hypothetical vignette (*n* = 2420).

	Number (%)		*p* value
	Lack of benefit (*n* = 1209)	Potential harm (*n* = 1211)	
*Demographic characteristics*			
Country of residence			
Australia	600 (49.6)	602 (49.7)	0.967
United States	609 (50.4)	609 (50.3)	
Gender			
Male	624 (51.6)	622 (51.4)	0.935
Female	582 (48.1)	587 (48.5)	
Another gender or not provided	3 (0.2)	2 (0.2)	
Education			
High school diploma or less	349 (28.9)	364 (30.1)	0.738
Some college, associate's or trade school	451 (37.3)	454 (37.5)	
Bachelor's degree or higher	409 (33.8)	393 (32.5)	
*Diabetes‐related characteristics*			
Ever diagnosed with prediabetes or diabetes	408 (33.7)	388 (32.0)	0.562
Current type of diabetes			
Prediabetes	116 (9.6)	105 (8.7)	0.918
Type 1	18 (1.5)	221 (18.2)	
Type 2	232 (19.2)	221 (18.2)	
Do not currently have diabetes	32 (2.6)	35 (2.9)	
Do not know type	10 (0.8)	11 (0.9)	
Current control of Type 1 and Type 2 diabetes			
Very well controlled	103 (8.5)	111 (9.2)	0.641
Somewhat well controlled	126 (10.4)	111 (9.2)	
Not very well‐controlled	17 (1.4)	13 (1.1)	
Not sure	4 (0.3)	2 (0.2)	
Diabetes medicine			
Current	250 (20.7)	253 (20.9)	0.836
Previous	30 (2.5)	34 (2.8)	
Never	816 (67.5)	821 (67.8)	
*Other health‐related characteristics*			
Support needed to manage medicine			
Minimal support	960 (79.4)	979 (80.8)	0.832
Occasional support	104 (8.6)	100 (8.3)	
Complete assistance	32 (2.6)	29 (2.4)	
Self‐reported health			
Excellent or very good	348 (23.8)	345 (28.5)	0.646
Good	528 (43.7)	550 (45.4)	
Fair or poor	333 (27.5)	316 (26.1)	
Health literacy			
Extremely	735 (60.8)	733 (60.5)	0.827
Quite a bit	323 (26.7)	334 (27.6)	
Somewhat or less	151 (12.5)	144 (11.9)	

	**Median (IQR)**		
Age	70 (67–74)	70 (67–74)	0.966
Total number of medicines	4 (2–7)	4 (2–7)	
Prescription medicines	3 (1–5)	3 (1–5)	0.317
OTC medicines and supplements	1 (0–3)	1 (0–3)	
